# Association between handgrip strength and symptoms of anxiety and depression among university students: A cross‐sectional study

**DOI:** 10.14814/phy2.71143

**Published:** 2026-10-15

**Authors:** Liyan Khadeeja, B. K. Manjunatha Goud, Rajani Dube, Subhranshu Sekhar Kar, Burhanuddin Murtaza Patanwala

**Affiliations:** ^1^ RAK College of Medical Sciences RAK Medical and Health Sciences University Ras Al Khaimah United Arab Emirates; ^2^ Department of Biochemistry, RAK College of Medical Sciences RAK Medical and Health Sciences University Ras Al Khaimah United Arab Emirates; ^3^ Department of Obstetrics and Gynaecology, RAKCOMS RAK Medical and Health Sciences University Ras Al Khaimah United Arab Emirates; ^4^ Department of Pediatrics, RAKCOMS RAK Medical and Health Sciences University Ras Al Khaimah United Arab Emirates

**Keywords:** anxiety, depression, hand grip dynamometer, hand grip strength, mental health

## Abstract

Handgrip strength is a simple and objective measure of muscle function, increasingly associated with physical and psychological health. However, data regarding its link to anxiety and depression in young adults remain limited. The aim of this study was to assess handgrip strength in healthy university students and examine its association with symptoms of anxiety and depression. This cross‐sectional study included 216 undergraduate students (mean age: 20.6 ± 4.7 years). Handgrip strength of each participant was measured using a digital hand dynamometer. Symptoms of anxiety and depression were assessed using the GAD‐7 (Generalized Anxiety Disorder‐7) and the PHQ‐9 (Patient Health Questionnaire‐9), respectively. Pearson correlations and independent sample *t*‐tests were used for statistical analysis. Handgrip strength showed a small but statistically significant negative correlation with PHQ‐9 (left hand: *r* = −0.148, *p* = 0.030) and GAD‐7 (left hand: *r* = −0.185, *p* = 0.006) scores. These associations were stronger in female participants. Anxiety and depression scores were strongly positively correlated (*r* = 0.705, *p* < 0.001). The results of this study indicate lower handgrip strength was associated with higher anxiety and depression symptoms in healthy university students, especially in female students. However, further longitudinal studies are needed to establish causality.

## 1 INTRODUCTION

Hand Grip Strength (HGS) is a widely used and reliable measure of upper limb strength and overall neuromuscular function (Lee, 2021). HGS is measured using a hand dynamometer and provides an objective assessment of muscle performance, demonstrating high reliability and validity when standardized procedures are used (Mathiowetz et al., 1984). Clinically, it is used to monitor rehabilitation outcomes, assess functional limitations, and evaluate the ability to return to work or daily activities (Leong et al., 2015; Taekema et al., 2010).

Beyond musculoskeletal health, decreased handgrip strength has been linked to adverse health effects, including cardiovascular diseases (Artero et al., 2012; Gubelmann et al., 2017; Lawman et al., 2016; Lee et al., 2016), metabolic disorders (Wen et al., 2022), and increased all‐cause mortality (Gale et al., 2007; Rantanen et al., 2003). Hence, muscle strength is now considered an important biomarker for overall physical health. Recently, growing attention has been directed towards a potential relationship between muscle strength and mental health outcomes.

Anxiety and depression are among the most common mental disorders worldwide. According to the World Health Organization (WHO), approximately 470 million people suffer from anxiety disorders and approximately 322 million people suffer from depression globally, making these conditions among the leading causes of disability and reduced quality of life (WHO, 2025a, 2025b). Anxiety is generally characterized by excessive and persistent worry, accompanied by physiological symptoms such as restlessness and autonomic arousal, while depression is characterized by persistent low mood, loss of interest or pleasure, and reduced motivation [DSM‐5] (American Psychiatric Association, 2013; Belmaker & Agam, 2008). Both conditions are associated with impaired daily functioning, reduced academic performance among students, and long‐term negative health consequences, particularly in young adults (Brenes, 2007; Mendlowicz & Stein, 2000; Wells et al., 1989).

Emerging studies suggest an inverse relationship between hand grip strength and mental health. Several large population studies and systematic reviews have shown that individuals with a lower grip strength are more likely to experience anxiety and depressive symptoms (Ashdown‐Franks et al., 2019; Brooks et al., 2018; Cabanas‐Sánchez et al., 2022; Duchowny et al., 2022; Fukumori et al., 2015; Ganipineni et al., 2023; Gu et al., 2021; Jiang et al., 2022; Kim, 2019; Marconcin et al., 2020; McDowell et al., 2018; Smith et al., 2019; Smith et al., 2022; Zheng et al., 2022). Despite this growing evidence highlighting the potential link between grip strength and mental health disorders, such as depression, most of the existing studies on this relationship focus mainly on middle‐aged or older populations. Thus, data on young adults, particularly students, remain relatively limited. Moreover, much of the current literature has primarily examined the association between HGS and depression, with fewer studies exploring anxiety symptoms. This highlights an important gap, considering the high prevalence of psychological distress among university students and young adults (Lakasing & Mirza, 2020).

Sex differences may influence the relationship between handgrip strength and mental health, potentially due to hormonal, psychosocial, and behavioral factors. In addition, males and females differ in muscle mass, strength, and physical activity patterns, all of which may influence the association between muscle strength and psychological wellbeing. However, evidence regarding whether this relationship differs by sex remains inconsistent, particularly in young adults, warranting further investigation.

Given this high prevalence of anxiety and depression in young adults, and the practicality of measuring HGS, as a quick and inexpensive tool, further research is warranted to investigate their relationship in this age group. Understanding whether muscle strength is associated with psychological wellbeing could help with early identification and preventative strategies.

Therefore, the aim of this study was to assess handgrip strength and examine its association with symptoms of Generalized Anxiety Disorder (GAD) and Major Depressive Disorder (MDD) in otherwise healthy university students. We hypothesized that lower grip strength would be associated with higher anxiety and depression scores, and expected the magnitude of this association to vary between the sexes.

## 2 METHODS

### 2.1 Ethical approval

This study was conducted at the RAK Medical and Health Sciences University, UAE, at the Physiology Laboratory, over a five‐month period. Ethical approval was obtained from the Institutional Review Board (RAKMHSU‐REC‐132‐2022/23‐UG‐M). All participants were informed of the purpose of the study; the experimental procedures and potential risks before written informed consent was obtained. The study conforms to the standards of utilizing human subjects in research as detailed in the *Declaration of Helsinki*.

### 2.2 Participants

A total of 216 undergraduate students, both males and females (138 females and 78 males), aged 18–24 years from RAK Medical and Health Sciences University, UAE, participated in this study. Participants were recruited in the classrooms during university hours after being informed about the study objectives and procedures and obtaining informed consent. The participants included were apparently healthy individuals with no known cardiac, neurological or other health conditions and no history of hand or wrist surgery within the previous 6 months. Participants currently diagnosed with and undergoing treatment for anxiety or depression were excluded from the study. An a priori power analysis was not performed; therefore all eligible students who met the inclusion criteria and consented during the recruitment period were included in the study.

### 2.3 Hand grip strength measurement

Hand grip strength of each participant was measured using a Jamar plus+ Digital hand dynamometer (Performance Health, USA) (Mathiowetz, 2002). The dynamometer handle was adjusted to each participant's hand size. Participants were seated upright with the shoulder adducted, elbow flexed at 90°, forearm in neutral position, and wrist maintained between 0° and 30° of extension (Härkönen et al., 1993). Three maximal voluntary contractions were recorded for each hand with adequate rest between trials. The highest value obtained for each hand was used for analysis, in accordance with standardized handgrip assessment protocols (Bohannon, 1997).

### 2.4 Assessment of anxiety and depression

Following the grip strength measurements, participants' anxiety symptoms were then assessed using the Generalized Anxiety Disorder‐7 scale (GAD‐7, Anxiety and Depression Association of America, ADAA), (Spitzer et al., 2006) and depressive symptoms were evaluated using the Patient Health Questionnaire‐9 (PHQ‐9), American Psychological Association (APA) (Spitzer et al., 2019). Both tools are validated, self‐administered questionnaires assessing symptoms of the participants over the preceding 2 weeks. Scoring was performed according to standardized guidelines (Kroenke et al., 2001).

### 2.5 Statistical analysis

Statistical analyses were performed using a commercially available software tool (IBM SPSS Statistics, Version 29). Descriptive statistics were used to summarize participant characteristics. Gender‐based differences were compared using independent sample *t*‐tests. Pearson correlation coefficients were used to assess associations between hand grip strength and mental health scores. All correlation analyses were unadjusted and were performed using the available study variables. Data are presented as means ± SD. A *p*‐value < 0.05 was considered statistically significant.

A post hoc power calculation was performed using the observed effect sizes, the available sample size, and an *α* level of 0.05 to assess the statistical power of the observed associations.

## 3 RESULTS

### 3.1 Overall results and correlation analysis of the study population

A total of 216 participants (138 females and 78 males) were included in the analysis. Participant characteristics, handgrip strength measurements, and mental health scores are summarized in Table 1.

**TABLE 1:** Descriptive characteristics, handgrip strength measurements, and mental health scores of the study population.

	*N*	Mean	SD
Age	216	20.62	4.66
HGS LEFT	216	26.77	10.33
HGS RIGHT	216	28.47	11.58
GAD‐7	216	6.38	4.88
PHQ‐9	216	7.44	5.75

Abbreviations: GAD‐7, Generalized Anxiety Disorder‐7; HGS, handgrip strength; *N*, number of participants; PHQ‐9, Patient Health Questionnaire‐9; SD, standard deviation.

Sex‐based comparison of study parameters showed that male participants had significantly higher left‐ and right‐hand grip strength compared with female participants (*p* < 0.001). Female participants had significantly higher GAD‐7 scores compared with males (*p* = 0.005), whereas no significant sex difference was observed in PHQ‐9 scores (*p* = 0.123) (Table 2).

**TABLE 2:** Comparison of handgrip strength and mental health scores between male and female participants.

Parameters	Male (78)		Female (138)		*p* value
	Mean	SD	Mean	SD	
HGS LEFT	36.43	9.41	21.31	5.85	0.000
HGS RIGHT	39.34	11.0 1	22.32	6.08	0.000
GAD‐7	4.99	4.18	7.17	5.08	0.005**
PHQ‐9	6.42	5.31	8.02	5.92	0.123

* *p* <0.05.

** *p* <0.01.

*** *p* <0.001.

Left‐hand grip strength demonstrated weak but significant negative correlations with GAD‐7 and PHQ‐9 scores, whereas right‐hand grip strength showed no significant associations with either mental health measure. A strong positive correlation was observed between GAD‐7 and PHQ‐9 scores (Table 3).

**TABLE 3:** Correlation between handgrip strength and mental health scores in the study population.

		HGS LEFT	HGS RIGHT	GAD‐7	PHQ‐9
HGS LEFT	Pearson Correlation	1	0.932**	−0.185**	−0.148*
	Sig. (2‐tailed)		0.000	0.006	0.030
HGS RIGHT	Pearson Correlation	0.932**	1	−0.131	−0.106
	Sig. (2‐tailed)	0.000		0.054	0.119
GAD‐7	Pearson Correlation	−0.185**	−0.131	1	0.705**
	Sig. (2‐tailed)	0.006	0.054		0.000
PHQ‐9	Pearson Correlation	−0.148*	−0.106	0.705**	1
	Sig. (2‐tailed)	0.030	0.119	0.000	

* *p* <0.05.

** *p* <0.01.

*** *p* <0.001.

The post hoc power analysis showed that statistical power varied according to the magnitude of the observed associations. For the overall study population, power was approximately 78.3% for the association between left‐hand HGS and GAD‐7 scores (*r* = −0.185) and 94.6% for the association between left‐hand HGS and PHQ‐9 scores (*r* = −0.238), whereas smaller observed correlations showed lower power. These findings indicate that the study had greater power to detect moderate associations but limited power for smaller effects, particularly among nonsignificant associations.

### 3.2 Correlation analysis in male participants

Among male participants, left‐ and right‐hand grip strength demonstrated a strong positive correlation, indicating a strong relationship between bilateral grip strength measurements. Neither left‐ nor right‐hand grip strength showed significant associations with GAD‐7 or PHQ‐9 scores. A strong positive correlation was observed between GAD‐7 and PHQ‐9 scores, indicating that higher anxiety symptoms were associated with higher depressive symptoms among male participants (Table 4).

**TABLE 4:** Correlation between handgrip strength and mental health scores among male participants (*n* = 78).

		HGS LEFT	HGS RIGHT	GAD‐7	PHQ‐9
HGS LEFT	Pearson Correlation	1	0.930**	0.074	0.117
	Sig. (2‐tailed)		0.000	0.521	0.308
HGS RIGHT	Pearson Correlation	0.930**	1	0.091	0.147
	Sig. (2‐tailed)	0.000		0.427	0.200
GAD‐7	Pearson Correlation	0.074	0.091	1	0.774**
	Sig. (2‐tailed)	0.521	0.427		0.000
PHQ‐9	Pearson Correlation	0.117	0.147	0.774**	1
	Sig. (2‐tailed)	0.308	0.200	0.000	

* *p* <0.05.

** *p* <0.01.

*** *p* <0.001.

### 3.3 Correlation analysis in female participants

Among female participants, left‐ and right‐hand grip strength demonstrated a strong positive correlation, indicating a strong relationship between bilateral grip strength measurements. Left‐hand grip strength showed a significant negative association with PHQ‐9 scores, while right‐hand grip strength demonstrated a smaller significant negative association with PHQ‐9 scores. Neither left‐ nor right‐hand grip strength showed significant associations with GAD‐7 scores. A strong positive correlation was observed between GAD‐7 and PHQ‐9 scores, indicating that higher anxiety symptoms were associated with higher depressive symptoms among female participants (Table 5).

**TABLE 5:** Correlation between handgrip strength and mental health scores among female participants (*n* = 138).

		HGS LEFT	HGS RIGHT	GAD‐7	PHQ‐9
HGS LEFT	Pearson Correlation	1	0.759**	−0.142	−0.238**
	Sig. (2‐tailed)		0.000	0.096	0.005
HGS RIGHT	Pearson Correlation	0.759**	1	−0.015	−0.168*
	Sig. (2‐tailed)	0.000		0.861	0.049
	Sig. (2‐tailed)	0.000	0.000	0.697	0.786
GAD‐7	Pearson Correlation	−0.142	−0.015	1	0.668**
	Sig. (2‐tailed)	0.096	0.861		0.000
PHQ‐9	Pearson Correlation	−0.238**	−0.168*	0.668**	1
	Sig. (2‐tailed)	0.005	0.049	0.000	

* *p* <0.05.

** *p* <0.01.

*** *p* <0.001.

## 4 DISCUSSION

In this study, we assessed the relationship between handgrip strength and symptoms of anxiety and depression in healthy university students. We observed a small but statistically significant inverse correlation between grip strength and mental health symptoms (Table 3 and Figure 1). Our findings are consistent with those reported in previous population‐based studies, such as Jiang et al. (2022), Duchowny et al. (2022), Ashdown‐Franks et al. (2019), and Gu et al. (2021), which showed similar associations across different populations. These studies suggest that lower grip strength is consistently associated with an increased risk of anxiety and depression. While most previous studies have focused primarily on middle‐aged and older‐aged populations, our study highlights that this relationship may also exist in younger populations (mean age 20.62 ± 4.66 years) as shown in our sample. Hence, this suggests that the link between muscle strength and mental health may emerge early in life and is not limited to aging populations.

**FIGURE 1:**
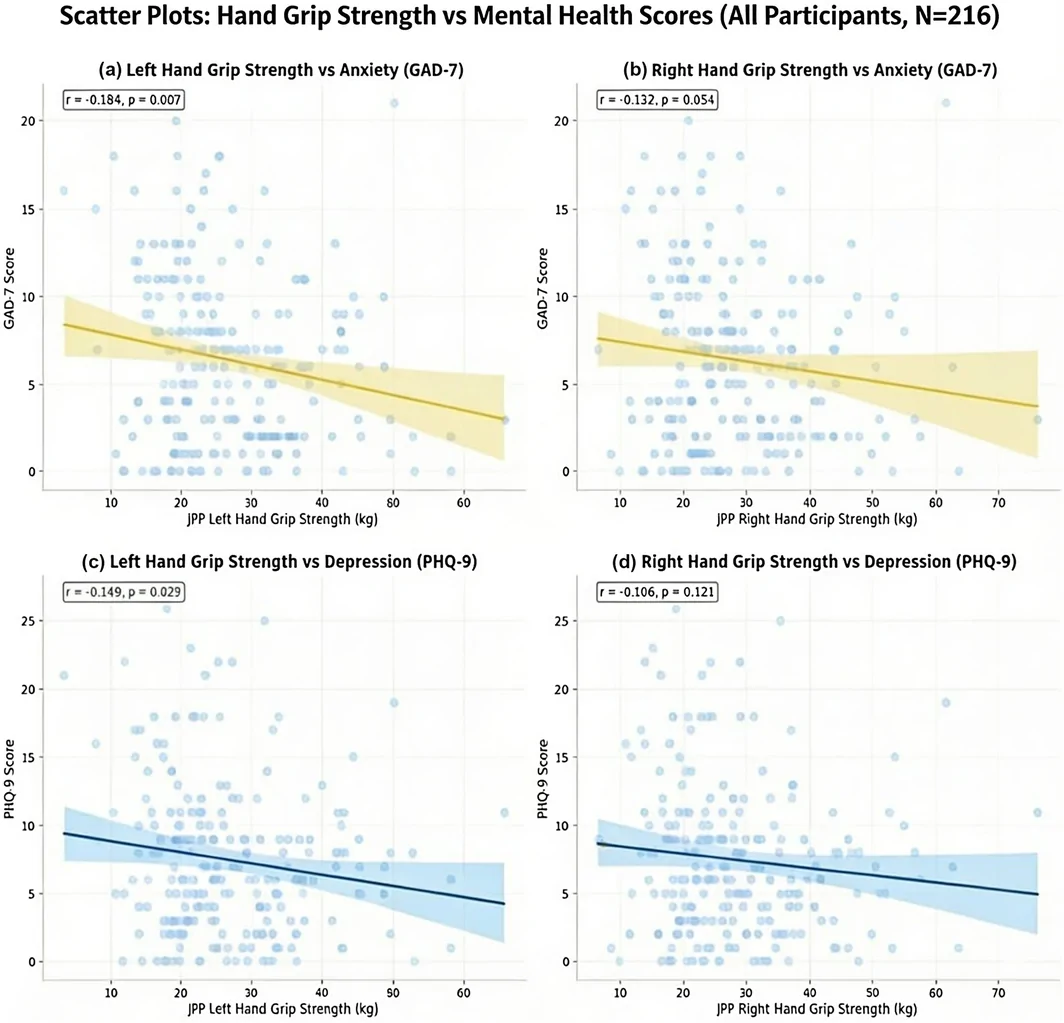
Scatter plots demonstrating the association between handgrip strength and mental health scores in the total study population.

Differences between right‐ and left‐hand grip strength were observed in our sample. Such bilateral variations may reflect differences in habitual hand use, individual hand preference, occupational or daily activities, and neuromuscular adaptation. However, as hand dominance was not assessed in this study, it is not possible to determine whether these differences correspond to dominant versus non‐dominant hand strength. Future studies should incorporate hand dominance assessment to better interpret variations in bilateral handgrip strength.

Sex differences in handgrip strength were observed in the present study, with male participants demonstrating significantly greater left‐ and right‐hand grip strength than female participants. This finding is consistent with previous studies reporting higher handgrip strength among males than females, including those by Smith et al. (2022), McDowell et al. (2018), and Puh (2010). These differences are likely to reflect established sex‐related differences in muscle mass and muscular strength (Janssen et al., 2000). However, because muscle mass and other anthropometric or physiological measures were not assessed in the present study, the specific factors underlying the observed differences in HGS cannot be determined.

The observed sex‐specific pattern in the associations between HGS and psychological symptoms may have several possible explanations. In the present study, female participants had significantly higher GAD‐7 scores than male participants, although no significant sex difference was observed in PHQ‐9 scores. Furthermore, among female participants, both left‐ and right‐hand HGS showed significant negative correlations with PHQ‐9 scores, whereas no significant associations between HGS and PHQ‐9 were observed among male participants. Neither HGS measure was significantly associated with GAD‐7 scores in females or males. These findings suggest that the association between HGS and psychological symptoms may differ according to sex and outcome measure, although the cross‐sectional design and limited sample size within each sex group preclude definitive conclusions regarding these differences.

Several biological and behavioral factors may potentially contribute to this pattern. Sex hormones have established roles in both skeletal muscle physiology and psychological processes. Testosterone contributes to muscle protein synthesis, muscle mass, and muscular strength, while estrogen may influence skeletal muscle metabolism, recovery, and neuromuscular function. Sex‐related differences in these physiological processes could therefore potentially contribute to differences in HGS and its relationship with psychological symptoms. However, hormone concentrations were not measured in the present study, and these mechanisms should therefore be regarded as plausible explanations rather than demonstrated pathways.

Differences in muscle mass and physical activity may also be relevant. Sex differences in anxiety and related psychological symptoms have been reported in previous research (Jalnapurkar et al., 2018; McLean et al., 2011). Women generally have lower absolute muscle mass and muscular strength than men, which may partly explain the lower HGS observed among female participants (Janssen et al., 2000). Differences in perceived stress and coping have also been observed among college students (Graves et al., 2021), while stress‐related biological and psychological processes may further contribute to poorer mental health outcomes (Daviu et al., 2019; Kim et al., 2022). Sex‐related differences in habitual physical activity have also been reported (Azevedo et al., 2007), and physical activity has been associated with both muscular strength and psychological well‐being (Barre et al., 2024; Labott et al., 2019; Mahindru et al., 2023). Nevertheless, physical activity, stress levels, and other relevant behavioral or psychosocial variables were not directly assessed in the present study and therefore could not be evaluated as potential confounding or explanatory factors.

Beyond sex‐specific factors, several biological and behavioral pathways have been proposed to explain the relationship between muscular strength and psychological health. Greater handgrip strength has been associated with markers of brain health, including hippocampal volume and white matter integrity, which are relevant to neurobiological processes involved in depression (Firth et al., 2020). However, these mechanisms were not directly assessed in the present study and should therefore be interpreted as hypotheses that may help explain the observed associations rather than as established causal pathways.

Overall, the findings indicate that lower HGS was associated with greater psychological symptom burden in the study population, with the observed associations differing by sex and psychological outcome. The underlying mechanisms remain uncertain. Future studies incorporating measures of body composition, physical activity, hormonal status, stress, sleep, and other relevant behavioral and psychosocial factors, preferably within larger and longitudinal cohorts, are warranted to determine whether these factors explain the observed sex‐specific patterns.

From a clinical perspective, handgrip strength is a simple, inexpensive, and non‐invasive measure that may warrant further investigation as a marker associated with psychological wellbeing in young adults. However, its potential clinical utility cannot be determined from the present cross‐sectional study and requires validation in larger longitudinal studies.

The cross‐sectional nature of this study does not allow the establishment of a causal relationship. It remains unclear whether reduced handgrip strength contributes to poorer mental health, whether psychological distress leads to reduced muscle strength, or whether both are influenced by shared underlying factors.

Other limitations include the assessment of anxiety and depression using validated self‐administered questionnaires without clinical diagnostic interviews, which may have introduced reporting bias. In addition, participants were recruited from a single institution, limiting the generalizability of the findings. Additionally, hand dominance was not recorded, which limited our ability to interpret whether observed differences between right and left handgrip strength reflected dominant versus non‐dominant hand differences. Several potential confounding variables, including anthropometric measures (such as height, weight, and body mass index), physical activity level, sleep quality, caffeine intake, socioeconomic status, and other lifestyle factors, were not collected as part of the study protocol. Therefore, multivariable adjustment for these factors was not performed, and their potential influence on the observed associations cannot be excluded.

The post hoc power estimates varied across the observed associations, reflecting the small effect sizes and the sample size available for the analyses. Therefore, the nonsignificant associations, particularly those involving right‐hand HGS, should be interpreted cautiously and cannot be taken as evidence of no association. Larger studies with prespecified sample‐size calculations are warranted.

Despite these limitations, this study adds to the growing body of evidence supporting an association between muscle strength and mental health (Kwak & Kim, 2022). Our findings suggest that this relationship may already be present in young, healthy adults. Future longitudinal studies are needed to clarify the direction and underlying mechanisms of this association and to determine its potential clinical relevance.

## 5 CONCLUSION

This study demonstrated that lower handgrip strength was associated with higher symptoms of anxiety and depression among healthy university students. These findings suggest that the relationship between muscle strength and mental health may extend to young adult populations. Further research is needed to clarify the mechanisms underlying this association and to determine its broader clinical significance.

## AUTHOR CONTRIBUTIONS

**Liyan Khadeeja:** Conceptualization; data curation; formal analysis; investigation; methodology; project administration; resources. **B. K. Manjunatha Goud:** Conceptualization; data curation; formal analysis; funding acquisition; project administration; resources; software; supervision; validation. **Rajani Dube:** Project administration; resources; software; supervision. **Subhranshu Sekhar Kar:** Project administration; resources; software; supervision. **Burhanuddin Murtaza Patanwala:** Validation; visualization.

## FUNDING INFORMATION

This research received no external funding.

## ETHICS STATEMENT

This study was conducted at the RAK Medical and Health Sciences University, UAE, at the Physiology Laboratory, over a five‐month period. Ethical approval was obtained from the Institutional Review Board (RAKMHSU‐REC‐132‐2022/23‐UG‐M). All participants were informed of the purpose of the study; the experimental procedures and potential risks before written informed consent was obtained. The study conforms to the standards of utilizing human subjects in research as detailed in the *Declaration of Helsinki*.

## Data Availability

The data supporting the findings of this study are available upon reasonable request.
